# Design, development and validation of a questionnaire to assess dentists’ knowledge and experience in diagnosing, recording, and managing root caries

**DOI:** 10.1007/s00784-022-04842-x

**Published:** 2023-01-11

**Authors:** Samira Helena Niemeyer, Sabrina Maniewicz, Guglielmo Campus, Christian Tennert, Burak Yilmaz, Alkisti Zekeridou, Andrea Roccuzzo, Marcella Esteves-Oliveira, Thiago S. Carvalho, Richard Johannes Wierichs

**Affiliations:** 1 Department of Restorative, Preventive and Pediatric Dentistry, University of Bern, zmk bern Freiburgstrasse 7, CH-3010 Bern, Switzerland; 2 Division of Gerodontology and Removable Prosthodontics, University Clinics of Dental Medicine, University of Geneva, Geneva, Switzerland; 3 Department of Surgery, Microsurgery and Medicine Sciences, School of Dentistry, University of Sassari, Sassari, Italy; 4 Department of Reconstructive Dentistry and Gerodontology, University of Bern, zmk bern, Bern, Switzerland; 5 Division of Regenerative Dentistry and Periodontology, University Clinics of Dental Medicine, University of Geneva, Geneva, Switzerland; 6 Department of Periodontology, School of Dental Medicine, University of Bern, Bern, Switzerland

**Keywords:** Root caries, Questionnaire, Validation, Management, Non-invasive treatment, Minimal-invasive treatment

## Abstract

**Objectives:**

The prevalence of root caries is increasing globally, especially in the elderly population, and even though the number of patients with root caries lesions is augmenting, there are still many discrepancies in how dentists manage this condition. The present study aimed to develop and validate a questionnaire to evaluate how dentists diagnose, record and manage root caries lesions, and to verify the validity and reliability of this questionnaire.

**Materials and methods:**

An expert panel developed a self-administered questionnaire survey with three domains: (1) dentists’ knowledge on diagnosis, recording, and managing root caries; (2) information about their current general clinical routines; (3) their demographics. The original English [E] version was translated into three different languages (French [*F*], German [*G*], Italian [*I*]), and subsequently back-translated into English by independent dentists. For the validation, 82 dentists (20–22 for each of the translated versions) accepted to answer the questionnaire at two different time-points (with 1-week interval). The data was quality checked. Construct validity, internal reliability, and intra-class correlation (ICC) were assessed.

**Results:**

Seventy-seven dentists completed the questionnaire twice [*E*: 17; *F*: 19; *G*: 19; *I*: 22]. The mean ICC (standard deviation) was 0.98(0.03) for *E*, 0.90(0.12) for *F*, 0.98 (0.04) for *G*, and 0.98 (0.01) for *I*. Overall, the test–retest reliability was excellent (mean ICC (SD): 0.96 (0.08)). Furthermore, the questionnaire demonstrated good internal reliability (inter-observer reliability; Fleiss kappa: overall:0.27(fair); *E*:0.30 (fair); *F*: 0.33(fair); *G*: 0.33(fair); *I*: 0.89 (almost perfect)).

**Conclusion:**

The questionnaire was validated and is suitable to be used in the four languages to assess the knowledge of dentists on diagnosing, recording and managing root caries.

**Clinical significance:**

The present questionnaire was validated and seems to be a good tool to evaluate how dentists diagnose, record, and manage root caries lesions both in its original (English) and its translated (French, German, and Italian) versions.

**Supplementary information:**

The online version contains supplementary material available at 10.1007/s00784-022-04842-x.

## Introduction

In recent decades, life expectancy has been gradually increasing in many countries, and this increase in age may bring along many health vulnerabilities to the elderly population. These include decreased motor skills, which lead to difficulties in performing proper oral hygiene [1, 2] and hyposalivation [3]. This may, in turn, increase the risk of developing oral health problems [4] such as higher indices of gingival recession and root exposure, thereby augmenting the prevalence of root caries. Consequently, a concern regarding this condition is growing, mainly linked to the discrepancies in how dentists manage root caries [5–7]. This is also concerned in the recommendation of the European Federation of Conservative Dentistry (EFCD) and American Dental Association (ADA) [5, 6, 8].

Several factors can influence the success rate of the management of root caries lesions, including non-invasive treatments for inactivation of non-cavitated carious lesions through monitoring and individualized oral health instructions [9, 10]. For invasive treatment of cavitated lesions, the extension of the restauration is a major determinant of clinical success, as those extending to the proximal area, or involving two surfaces, have failure rates twice as high than those of single-surface restorations [10]. The higher frequency of checkup examinations, namely more than twice a year, is also a factor influencing the treatment of these lesions, as it increases the intervention rates on inactive lesions, leading to treatments where no direct intervention would be necessary [9].

Furthermore, the wide range of non-invasive and invasive strategies to prevent, arrest, or treat root caries lesions can lead to discrepancies in how dentists manage this condition [5–7, 11]. However, there is currently almost no information on how these professionals are managing root caries in their private practices. Until now, only two studies, one in Germany [9, 10] and the other in Queensland, Australia [7], have assessed these factors to some degree, but there is still a lack of consistency regarding risk assessment of root caries lesions. Overall, all studies suggested a need for improving the recording systems, as almost a quarter of the dentists in Queensland reported not being able to easily differentiate root from coronal caries in the patient files [7].

In this regard, it is important to further investigate how dentists deal with root caries, including their attitudes, difficulties, and opinions on the efficacy of treatment. Considering that well-designed questionnaires facilitate easy collection of data from participants in private clinics [12], the present study was designed to develop and validate a questionnaire to evaluate how dentists diagnose, record, and manage root caries lesions, and to verify the validity and reliability of this questionnaire.

## Materials and methods

### Development and standardization of the questionnaire

**Fig. 1:**
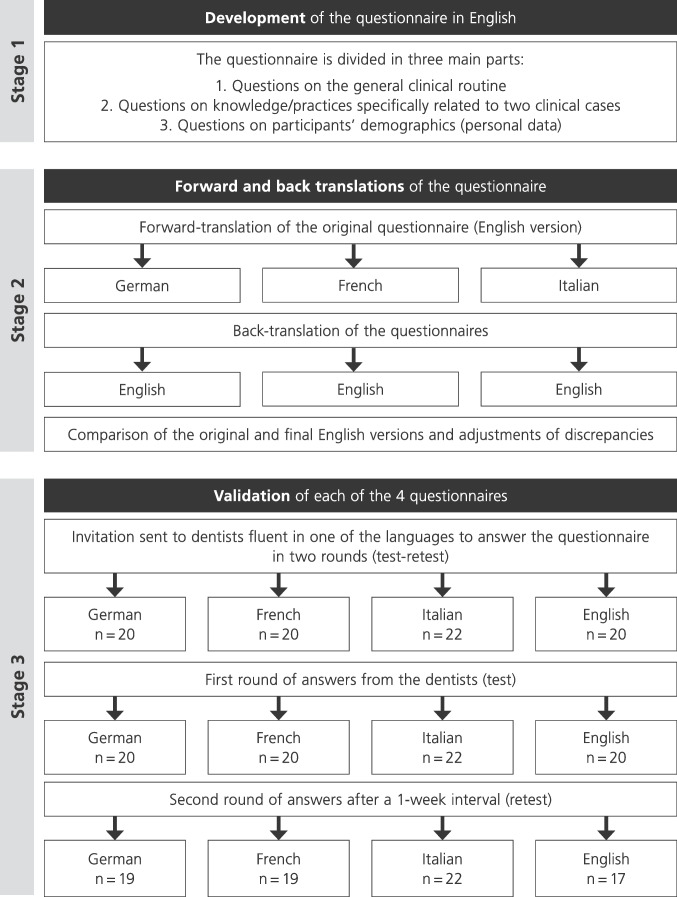
Schematic workflow of the development and refinement of the questionnaire

The process included several stages [13]: Stage 1: Construction of the questionnaire; Stage 2: Adaptation of the questionnaire; Stage 3: Pilot test and semantic adjustment of the questionnaire (Fig. 1).

Stage 1: Construction of the questionnaire

An international panel of experts (SHN, SM, GC, TS, RJW) was set to design and create a questionnaire based on relevant points regarding root caries and on previous surveys in cariology [7, 14, 15]. The questionnaire consisted of 9 questions including two clinical cases and was constructed in English. The experts discussed the most important points to be addressed in the questionnaire and the best way of wording and formulating the questions, covering all relevant points of root caries, namely regarding methods of diagnostics, record, and management. Additionally, the clinical cases were verified considering the inclusion of the most relevant scenarios concerning root caries lesions in older patients.

The questionnaire contained closed-ended questions, with a combination of multiple-choice and checkbox questions, as well as Likert scales. These types of questions take less time to answer, helping to increase the response rates, and favoring the quantitative analysis of the responses. The questionnaire was structed in three parts: (1) questions on the dentists’ general clinical routine (e.g., what kind of patients are treated in their practice, including information on their diagnostic methods and documentation of findings); (2) questions on the dentists’ knowledge/practices, specifically regarding two clinical cases containing clinical pictures, x-rays, and other necessary information about the patient (e.g., the dentists’ views on diagnosis, recording, and management of root caries is evaluated); (3) questions on the dentists’ demographics (e.g., year and place of graduation, specialization/area of practice, location of work).

The expected average time of completion of the questionnaire was set at 15 min. Furthermore, some questions in the questionnaire contained answers that were not scientifically justified, but were intentionally inserted to verify that answers were selected solely based on a certain logic (e.g., visual appearance in question 2 or some of the risk factors in question 5).

Stage 2: Adaptation of the questionnaire

Forward translation of the questionnaire from the original English version into the different languages (French, German, and Italian) was performed by clinicians actively involved in caries research whose native language were either French, German, or Italian and who also were competent in English (SHN, SM, GC, RJW). To ensure the accuracy of the forward translation, the questionnaire was later back-translated to English by other independent translators (who were not involved in the forward translations) (GC, CT, BY, AZ, AR, ME-O, TSC). The original and final English versions were compared, and any major discrepancies were assessed by the panel of experts and subsequently adjusted. In case of adjustments, the translated versions were modified accordingly.

Stage 3: Pilot test and semantic adjustment of the questionnaire

The different versions of the questionnaire were validated for their use. Switzerland was chosen as it has four official languages and the questionnaire would be available in, at least, the three most spoken/used languages of the country (French, German, and Italian) and English.

For each language, a sample of 20 dentists, native speakers in the respective language, were asked to answer the respective questionnaire.

The translated questionnaire was administered to the pilot sample, using the REDCap platform. To determine its reliability, the same dentists had to answer the same questionnaire 7 days after the first administration, without any recommendations or feedback from the researchers.

### Study setting/population

The target population of the survey were dentists actively working in national health systems and in private or public clinics, including general and specialist dentists. For the validation of the questionnaire in all four languages, we invited dentists actively working at universities in Switzerland (French, German), Italy (Italian), and the USA (English).

### Ethical aspects

The participation of the dentists was voluntary. They were informed about the study and the privacy of their data. Privacy was obtained by using the REDCap platform, which allows for password-protected, anonymous answers, keeping the identity of the subjects blinded. The dentists were asked to answer questions on demographics, but their identities were neither requested nor revealed. Individual responses were also not of interest, but rather the collective and combined outcomes derived from each participant at an aggregate level.

According to the European Guidelines for Good Clinical Practice (CPMP/ICH/135/95) and the Ethics Committee of the Canton of Bern, no approval by the local ethics committee was required. In any case, an informed consent question was embedded on the first page of the questionnaire, and if the participants answered “YES” to this question, they agreed to participate in the study and were automatically directed to the survey questions [13].

### Sample size calculation

For the validation process, the volunteer dentists had to answer the same questionnaire (same language version) at two different moments, 1 week apart. The test–retest reliability would be calculated with intra-class correlations (ICC), where we considered a minimum acceptable reliability (*ρ*0) of 0.7 and hoped reliability (*ρ*1) of 0.9. Using these parameters, the calculated sample size was 17 dentists per version of the questionnaire. Considering a drop-out rate of 10%, a minimum of 20 dentists were invited for each of the language versions of the questionnaire (English, French, German, and Italian), totaling a minimum of 80 dentists.

### Data management and statistical analysis

The questionnaire was analyzed as done previously [14]. For this, the data of the questionnaire was organized by using a databank and statistical analysis was performed with IBM SPSS 26. For comparison of back-translated versions and the English original questionnaire the plagiarism software provided by the University of Bern (www.plagscan.com) was used. This way, synonyms were uncovered that may have led to a change in meaning during forward- and back translation.

For the validation of the questionnaire, the degree of agreement among independent observers (Fleiss kappa coefficients) and the description of how strongly units in the same group resemble each other (intra-class correlation coefficients) were used to assess test–retest reliability (values above 0.6 was deemed as moderate to substantial agreement) [16]. To analyze the level of (dis-)agreement between dentists of the present cohort in diagnosing root caries, question 2 was analyzed separately using fleiss kappa coefficients.

## Results

The different versions of the questionnaire can be found in the supplementary document. After back translation of the forward-translated versions, the agreement with the original English questionnaire was 93% for the French, 88% for the German, and 71% for the Italian version. The content of the questionnaires was not altered in any of the versions.

Five dentists did not complete the questionnaire the second time around (English (*n* = 3), French (*n* = 1), German (*n* = 1)]), whereas 77 dentists completed the questionnaire twice (English (*n* = 17), French (*n* = 19), German (*n* = 19), Italian (*n* = 22)). They answered the questionnaire completely and there were no missing values.

The overall mean (standard deviation) intra-class correlation coefficient between baseline and second administration of the questionnaire (*n* = 77) was excellent at 0.96 (0.08). Considering the individual versions of the questionnaire, the ICC was 0.98 (0.03) for English, 0.90 (0.12) for French, 0.98 (0.04) for German, and 0.98 (0.01) for Italian (Table 1). Furthermore, the questionnaire demonstrated good internal reliability [inter-observer reliability; Fleiss kappa: overall: 0.27 (fair); English 0.30 (fair); French: 0.33 (fair); German: 0.33 (fair); Italian: 0.89 (almost perfect)].

**Table 1:** Overall intra- and inter-observer test reliability

Questionnaire		*n*	Result	SD	Interpretation
All languages	Mean ICC	77	0.96	0.08	Excellent
	Fleiss kappa	77	0.27	n/a	Fair
English	Mean ICC	17	0.98	0.03	Excellent
	Fleiss kappa	17	0.30	n/a	Fair
French	Mean ICC	19	0.90	0.12	Excellent
	Fleiss kappa	19	0.33	n/a	Fair
German	Mean ICC	19	0.98	0.04	Excellent
	Fleiss kappa	19	0.33	n/a	Fair
Italian	Mean ICC	22	0.98	0.01	Excellent
	Fleiss kappa	22	0.89	n/a	Almost perfect

For intra-observer test–retest reliability mean (SD = standard deviation), the mean intra-class correlations (ICC) of all participants is shown. For inter-observer reliability, the Fleiss kappa values are presented. Results are presented with and without separating between the four languages

Regarding the diagnosis of active and inactive root caries lesions, the intra-class correlation coefficient was excellent at 0.92 (0.14) (English 0.91 (0.15); French: 0.92 (0.12); German: 0.88 (0.03); Italian: 0.98 (0.03)) (Table 2). However, inter-observer reliability varied between 0.18 and 0.54 (Table 2).

**Table 2:** Intra- and inter-observer test reliability of criteria to discriminate between active and inactive root caries (question 2)

Questionnaire		*n*	Result	SD	Interpretation
All languages	Mean ICC	77	0.92	0.14	Excellent
	Fleiss kappa	77	0.17	n/a	Slight
English	Mean ICC	17	0.91	0.15	Excellent
	Fleiss kappa	17	0.18	n/a	Slight
French	Mean ICC	19	0.92	0.12	Excellent
	Fleiss kappa	19	0.27	n/a	Fair
German	Mean ICC	19	0.88	0.03	Excellent
	Fleiss kappa	19	0.27	n/a	Fair
Italian	Mean ICC	22	0.98	0.03	Excellent
	Fleiss kappa	22	0.54	n/a	Moderate

For intra-observer test–retest reliability mean (SD = standard deviation), the mean intra-class correlations (ICC) of all participants is shown. For inter-observer reliability, the Fleiss kappa values are presented. Results are presented with and without separating between the four languages

## Discussion

To the best of our knowledge, this is the second study presenting a questionnaire to evaluate dentists’ knowledge as well as their detection and treatment decisions related to root caries management. Our current questionnaire was validated in four languages: English, French, German, and Italian, showing an excellent inter-rater reliability, with the overall intra-class correlation coefficient of 96%. Even when considering each language individually, the ICC was 90% or above.

This excellent test–retest reliability is in agreement with previous validation processes, which were carried out for a malocclusion impact scale for early childhood [17], an Italian version of the Oral Health Impact Profile-14 [16], and a survey among dentists on attitudes and behavior regarding dentin caries removal [14]. However, in contrast to these previous studies, the inter-observer reliability showed only a fair overall value of 27%, differing between the languages. An almost perfect reliability was observed for the Italian version, whereas only a fair reliability could be observed for the English, French, and German versions. This difference occurred potentially because the Italian-speaking dentists were selected from a postgraduate education program, who had recently received a lecture on non-invasive and minimal-invasive therapies of root caries. This lecture was not a part of our validation process, so it was not performed for the English-, French-, and German-speaking dentists. The fair Fleiss kappa agreement for at least three of four languages could be interpreted as poorly validated questionnaire. However, it is more likely that the disparity in the inter-observer reliability of the different language versions of the questionnaire highlights three points: firstly, there are clear discrepancies in how dentists diagnose and manage root caries—even in such an apparently homogenous group of dentists; secondly, there is major need for educational courses regarding root caries treatment; and thirdly, the (short-term) impact of one lecture on treatment decisions. Accordingly, the questionnaire does seem to be a good measurement of the dentists’ knowledge on root caries.

The questionnaire is made up of only 9 questions: 6 related to dentists’ knowledge on root caries, 2 on clinical decision making, and 1 on dentists’ demographics. Our validation shows that this short form can be considered as an adequate tool to obtain the necessary data to draw the conclusions related to our aims [16]. Moreover, short questionnaires are also a more efficient way of data collection based on the premise that long questionnaires cannot be used in some research settings and private clinics [16]. Answering long questionnaires may not be useful due to the burden placed on patients and clinicians, even though it would presumably provide more comprehensive data [18].

When using surveys, mostly subjective outcomes are used. Personal beliefs might influence the data and the results might not reflect daily dental care. This could be a sign of a cognitive dissonance, where the participants’ answers might not mirror their actions when confronted with the clinical situation. This influence might be reduced by using clinical data [16]. Therefore, we included questions regarding two clinical cases, which were classified by the expert panel as common situations in the treatment of root caries. These quasi-practical questions were also crucial to observe the differences between the dentists who were introduced to the lecture and those who were not.

In the present study, the number of participants was chosen to validate the questionnaire not to present first result on the questions asked per se. Consequently, only the results on the validation process are presented, but not the results on the questions. Furthermore, the dentists involved in this validation all work at universities and are, thus, a homogenous group of dentists, albeit not a representative sample of dentists in the whole population. Further studies are already planned with representative samples from different countries in Western Europe. Nevertheless, the present investigation offers evidence about the convergent validity, discriminant validity, internal consistency, and test–retest reliability of the questionnaire.

In conclusion, the questionnaire showed not only an overall excellent test–retest reliability but also for each of the languages when analyzed separately. This indicates that it is valid and reliable, and it can be used in all four languages in which it was validated. Since excellent test–retest reliability could also be seen when the questions were considered alone the questionnaire is a valuable and suitable instrument for assessing the methods of diagnosing, recording and managing root caries among dentists.

## Supplementary information

Below is the link to the electronic supplementary material.

Supplementary file1 (PDF 2462 KB)

## Data Availability

All data generated or analyzed during this study are included in this article (and/or) its supplementary material files. Further enquiries can be directed to the corresponding author.
