# Supplementary material for: Design, development and validation of a questionnaire to assess dentists’ knowledge and experience in diagnosing, recording, and managing root caries
Source: Clin Oral Investig. 2023 Jan 11;27(6):2705–11. doi: 10.1007/s00784-022-04842-x (PMC10264516; doi:10.1007/s00784-022-04842-x)
Supplement: Supplementary file 1 — Supplementary file1 (PDF 2462 KB) [file 784_2022_4842_MOESM1_ESM.pdf]

u^b

UNIVERSITÄT BERN

zmk bern

Zahnmedizinische Kliniken der Universität Bern

AAA

English

Diagnosis and treatment decisions for root caries: a survey among dentists in Switzerland

Deutsch | English | Français | Italiano | Português

Information concerning the project

The elderly population is increasing and special attention to their oral health should be taken. One major problem in this population is root caries. Since the number of people over the age of 60 years is expected to rise, root caries is a condition with a growing prevalence. In recent years several methods to non- and invasively manage root caries lesions have been discussed. Therefore, the results of the present questionnaire will be used to firstly collect the actual state of the art in the management of root caries and to improve the management of root caries.

General information concerning the questionnaire

Thank you for taking some minutes to answer and send this questionnaire. It contains nine questions concerning the assessment of the diagnostics, recording and management of root caries lesions. Please answer the questions by ticking. It will take about fifteen minutes to completely answer the questionnaire. Your answers will be evaluated anonymously and data will be erased according to data protection guidelines. Many thanks for your cooperation!

Question 1

Do you have patients with root caries?

- ○ Yes, frequently (> 5 Patients average/week)
- ○ Yes, sometimes (3-5 Patients average/week)
- ○ Yes, rarely (1-2 Patients average/week)
- ○ Yes, almost never (< 1 Patients average/week)
- ○ Never

reset

Question 2

When discriminating between <u>active and inactive root caries lesions</u> which criteria do you use <u>to diagnose active ones</u>? (one or more answers for each criterion is permitted)

Color:

- ☐ Whitish surface
- ☐ Yellowish or light brown surface
- ☐ Dark brown to black surface
- ☐ Color is not a predominant criterion when discriminating between active and inactive root caries lesions

Visual appearance:

- ☐ Matt
- ☐ Shiny
- ☐ Visual appearance is not a predominant criterion when discriminating between active and inactive root caries lesions

Tactile sensation:

- ☐ Soft or leathery on probing
- ☐ Very soft on probing
- ☐ Hard on probing
- ☐ Tactile sensation is not a predominant criterion when discriminating between active and inactive root caries lesions

Cavitation:

- ☐ Presence of cavitation
- ☐ Absence of cavitation
- ☐ Cavitation is not a predominant criterion when discriminating between active and inactive root caries lesions

Location:

- ☐ Close to the gingival margin
- ☐ Distant from the gingival margin
- ☐ Close to the cement-enamel junction
- ☐ Areas of biofilm accumulation are more important than the distance to the gingival margin and to the cement-enamel junction
- ☐ Location is not a predominant criterion when discriminating between active and inactive root caries lesions

Do you use any other criteria? If yes, please specify: ____________________

Question 3

Do you use any additional method to assist in the diagnosis of root caries lesions (besides probe, mirror, tweezers, optical magnifier)?

- ○ No
- ○ Yes

reset

Please specify:

Question 4

Do you record root caries lesions in a way that distinguishes them from coronal caries lesions?

- ◉ No
- ○ Yes

reset

What is the reason for that?

- ☐ I do not think it is necessary/important
- ☐ My charting system does not allow
- ☐ Other

Do you record root caries lesions in a way that distinguishes them from coronal caries lesions?

- ○ No
- ◉ Yes

reset

Where do you record the root caries lesions?

- ○ In the (electronic) odontogram/ dental chart
- ○ In the (electronic) patient file
- ○ In both

reset

Question 5

Risk factors associated with root caries.
The following factors are important for a patient with exposed root surface to develop root caries. Do you agree or disagree?

	strongly disagree	disagree	agree	strongly agree	
**Existing untreated coronal caries**	○	○	○	○	reset
**Medication**	○	○	○	○	reset
**Poor oral hygiene**	○	○	○	○	reset
**Presence of periodontitis**	○	○	○	○	reset
**Presence of restored surfaces**	○	○	○	○	reset
**Root canal treatment**	○	○	○	○	reset

(Low) salivary flow rate

reset

High amount of S mutans in saliva

reset

Smoking

reset

Uncontrolled Diabetes

reset

Presence of plaque (or biofilm) at the root surfaces to be assessed

reset

Question 6

How do you feel when diagnosing root caries lesion?

- ○ Very confident
- ○ Confident
- ○ Uncertain
- ○ Very uncertain

reset

Question 7

Clinical Case A

Please reply to the questions below based on the following information and the pictures shown.

Patient: a 79 year-old male with compromised motor skills.

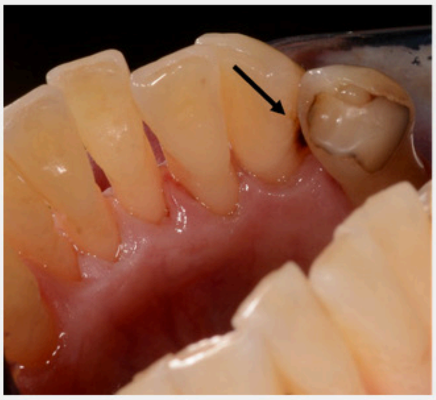

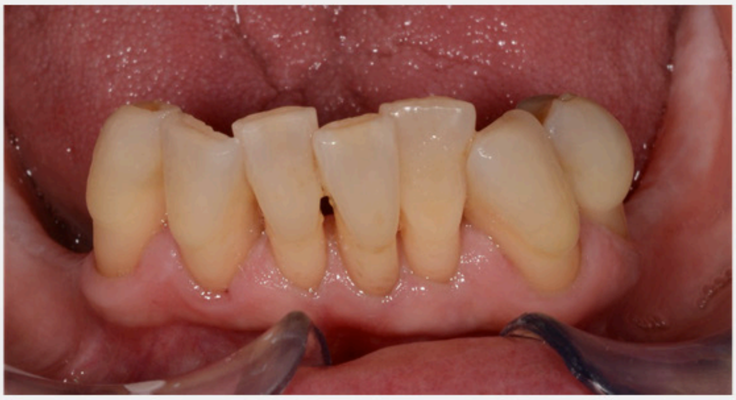

In your opinion, the marked root caries lesion on this tooth is:

- ○ Active
- ○ Inactive

reset

In your clinical routine, do you use any of the following treatment options for such lesions? (more than one option is possible)

- ☐ Instruction to improve the oral hygiene at home
- ☐ Advise to regularly use 1450 ppm of fluoride toothpaste at home

- ☐ Advise to regularly use 5000 ppm of fluoride toothpaste at home
- ☐ In-office biofilm removal
- ☐ In-office root scaling
- ☐ In-office application of chlorhexidine varnish
- ☐ In-office application of 38% silver diamine fluoride solution (approx. 35.400 ppm of fluoride)
- ☐ In-office application of 5% fluoride varnish (approx. 23.000 ppm of fluoride)

Would you restore such a lesion?

- ○ No
- ○ Yes

reset

If yes, which method to remove root caries do you use:

- ○ Complete caries excavation
- ○ Selective caries excavation
- ○ No caries excavation

reset

If yes, how would you access the lesion?

- ○ With slot preparation (occluso-approximal)
- ○ With slot preparation (vestibulo-approximal)
- ○ With atraumatic restorative treatment (ART)

If yes, which of the following materials/restorations would you use to restore the lesions?

	never	almost never	almost always	always	
**Amalgam**	○	○	○	○	reset
**Compomer**	○	○	○	○	reset
**Resin composite**	○	○	○	○	reset
**Flowable composite**	○	○	○	○	reset
**Crown (any material)**	○	○	○	○	reset
**Glass Ionomer Cement (GIC)**	○	○	○	○	reset
**Resin modified GIC**	○	○	○	○	reset

If yes, regarding the restorative materials, what would you expect for a 2-year success rate? (success = no further therapy necessary)

	0-20%	21-40%	41-60%	61-80%	81-100%	I don't know
Amalgam	○	○	○	○	○	○ reset
Compomer	○	○	○	○	○	○ reset
Resin composite	○	○	○	○	○	○ reset
Flowable composite	○	○	○	○	○	○ reset
Crown (any material)	○	○	○	○	○	○ reset
Glass Ionomer Cement (GIC)	○	○	○	○	○	○ reset
Resin modified GIC	○	○	○	○	○	○ reset

Do you have a recall program for this kind of patient in your practice?

- ○ No
- ○ Yes

reset

What recall interval do you suggest for this kind of patient?

- ○ None
- ○ Weekly
- ○ Monthly
- ○ Every 3 months
- ○ Every 6 months
- ○ Yearly
- ○ Two years or longer

reset

Question 8

Clinical Case B

Please reply to the questions below based on the following information and pictures shown.

Patient: 68 year-old female with good general health, controlled diabetes by continual use of medication.

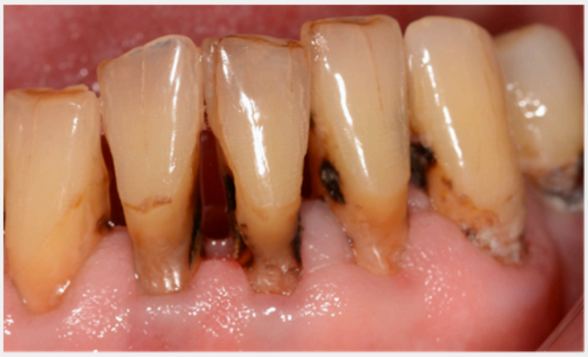
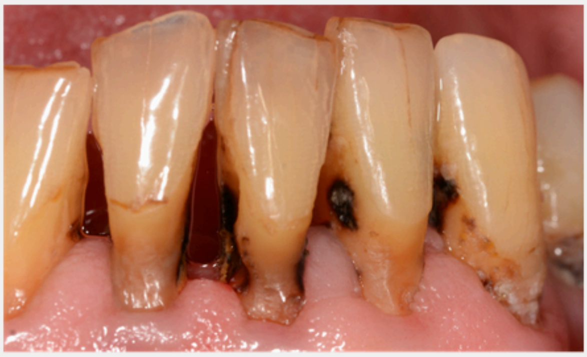

In your opinion, the marked root caries lesion on this tooth is:

- ○ Active
- ○ Inactive

reset

In your clinical routine, do you use any of the following treatment options for such lesions? (more than one option is possible)

- ☐ Instruction to improve the oral hygiene at home
- ☐ Advise to regularly use 1450 ppm of fluoride toothpaste at home
- ☐ Advise to regularly use 5000 ppm of fluoride toothpaste at home
- ☐ In-office biofilm removal
- ☐ In-office root scaling
- ☐ In-office application of chlorhexidine varnish
- ☐ In-office application of 38% silver diamine fluoride solution (approx. 35.400 ppm of fluoride)
- ☐ In-office application of 5% fluoride varnish (approx. 23.000 ppm of fluoride)

Would you restore such a lesion?

- ○ No
- ○ Yes

reset

If yes, which method to remove root caries do you use:

- ○ Complete caries excavation
- ○ Selective caries excavation
- ○ No caries excavation

reset

If yes, how would you access the lesion?

- ○ With slot preparation (occluso-approximal)
- ○ With slot preparation (vestibulo-approximal)
- ○ With atraumatic restorative treatment (ART)

reset

If yes, which of the following materials/restorations would you use to restore the lesions?

	never	almost never	almost always	always	
**Amalgam**	○	○	○	○	reset
**Compomer**	○	○	○	○	reset
**Resin composite**	○	○	○	○	reset
**Flowable composite**	○	○	○	○	reset
**Crown (any material)**	○	○	○	○	reset
**Glass Ionomer Cement (GIC)**	○	○	○	○	reset
**Resin modified GIC**	○	○	○	○	reset

If yes, regarding the restorative materials, what would you expect for a 2-year success rate? (success = no further therapy necessary)

	0-20%	21-40%	41-60%	61-80%	81-100%	I don't know	
**Amalgam**	○	○	○	○	○	○	reset
**Compomer**	○	○	○	○	○	○	reset
**Resin composite**	○	○	○	○	○	○	reset
**Flowable composite**	○	○	○	○	○	○	reset
**Crown (any material)**	○	○	○	○	○	○	reset
**Glass Ionomer Composite (GIC)**	○	○	○	○	○	○	reset
**Resin modified GIC**	○	○	○	○	○	○	reset

Do you have a recall program for this kind of patient in your practice?

- ○ No
- ○ Yes

reset

What recall interval do you suggest for this kind of patient?

- ◯ None
- ◯ Weekly
- ◯ Monthly
- ◯ Every 3 months
- ◯ Every 6 months
- ◯ Yearly
- ◯ Two years or longer

reset

Question 9

General Information

Please, provide information about you and your current working situation.

Year of birth: ______

Gender:

- ◯ Male
- ◯ Female

reset

Year of graduation from dental school: ______

Which University:

- ◯ Basel
- ◯ Bern
- ◯ Geneva
- ◯ Zurich
- ◯ Other

reset

Canton where you work: [dropdown]

Practice setting:

- ◯ City
- ◯ Town
- ◯ Rural area

reset

Type of practice:

- ◯ Private practice (1 dentist)
- ◯ Private practice (2 dentists)
- ◯ Private practice (>2 dentists)
- ◯ University
- ◯ (Dental) Hospital
- ◯ Defence Forces
- ◯ Dental Chain

reset

Estimated patient care hours per week:

- ○ 0-2h
- ○ 2-5h
- ○ 5-10h
- ○ 10-20h
- ○ 20-30h
- ○ 30-40h

reset

What is/are the main area(s) of your clinical practice? (more than one option is possible)

- ☐ General dentistry
- ☐ Oral Surgery
- ☐ Prosthodontics
- ☐ Prevention
- ☐ Endodontics
- ☐ Pediatrics
- ☐ Orthodontics
- ☐ Periodontology
- ☐ Aesthetic Dentistry
- ☐ Other

How do you keep yourself up-to-date in the field of prevention (more than one option is possible)?

- ☐ Post-graduate program
- ☐ Scientific articles
- ☐ Textbooks
- ☐ Congress/Symposium
- ☐ Guidelines from various dental associations
- ☐ I refer prevention cases to a hygienist. I, thus, do not need to be up to date in this area.

Submit
